# Role of Nonsteroidal Anti-Inflammatory Drugs (NSAIDs) in Cancer Prevention and Cancer Promotion

**DOI:** 10.1155/2019/3418975

**Published:** 2019-01-31

**Authors:** Rebecca S. Y. Wong

**Affiliations:** Faculty of Medicine, SEGi University, No. 9, Jalan Teknologi, Taman Sains Selangor, Kota Damansara, PJU 5, 47810 Petaling Jaya, Selangor, Malaysia

## Abstract

The nonsteroidal anti-inflammatory drugs (NSAIDs) are commonly prescribed by medical practitioners in many clinical conditions for the symptomatic treatment of pain and fever. Due to their anti-inflammatory properties, these drugs have been investigated for their anticancer effects in numerous studies. This is because chronic inflammation has long been linked to carcinogenesis. As such, anti-inflammatory drugs are believed to play a role in cancer treatment and prevention. In the past few decades, research has shown that NSAIDs may decrease the risk of certain types of cancer. However, there is also a growing body of research that proves the contrary. Furthermore, NSAIDs are well known for many side effects, including some life-threatening ones. This review will discuss the relationship between chronic inflammation and cancer, the role of NSAIDs in cancer prevention and cancer promotion, and some of the potentially lethal side effects of these drugs.

## 1. Introduction

The nonsteroidal anti-inflammatory drugs (NSAIDs) are among the most commonly prescribed medications worldwide. They consist of a group of drugs that are used in fever, pain, and inflammation because these drugs possess antipyretic, analgesic, and anti-inflammatory properties. Clinically, they are useful in relieving pain in many conditions, ranging from menstrual and postoperative pain to arthritic pain. These drugs are well-known anti-inflammatory agents, and they exert their effects through the inhibition of prostaglandin synthesis by blocking the enzyme cyclooxygenase (COX) [1]. In the past few decades, there is a growing body of research on the use of NSAIDs in cancer treatment and prevention, whereas the relationship between chronic inflammation and cancer has long been discovered [2].

There are numerous reports concerning the cancer-protective effects of NSAIDs in the published literature. Many of these studies are epidemiologic in nature, in which these drugs have been associated with a reduced cancer risk in various types of cancer such as breast [3–5], prostate [6, 7], colorectal [8, 9], ovarian [10], and head and neck cancers [11]. However, the role of NSAIDs in cancer prevention remains unclear due to contradicting and inconsistent findings. While some studies revealed a reduction in cancer risk, others demonstrated no association between cancer and NSAID use. For example, in a prospective study on about 20,000 women (aged 58–76 years), it was shown that nonaspirin NSAIDs were associated with neither ovarian nor uterine cancer risk [12].

The well-known anti-inflammatory effects of NSAIDs are one possible explanation for researchers' interest in their use in cancer prevention, as research has shown that many cancers are linked to inflammation [13]. It is, therefore, logical to believe that drugs that inhibit inflammation may be beneficial in cancer treatment or prevention. Other than their anti-inflammatory properties, some possible mechanisms which may play a role in the anticancer effects of NSAIDs include their ability to induce apoptosis, inhibit angiogenesis, and enhance cellular immune responses [14].

However, the relationship between cancer and NSAID use is complex and the inference that drugs which exert anti-inflammatory effects are also cancer protective is undoubtedly, an oversimplification. Previous studies have shown that the use of NSAIDs is associated with an increased risk or mortality in certain types of cancer [15, 16]. In addition, the long-term NSAID use is often associated with many serious cardiovascular, gastrointestinal, renal, and other side effects [17]. In view of these conflicting findings on the role of NSAIDs in cancer, this review will give an overview of the association between cancer and inflammation and the role of NSAIDs in cancer, in general. It will also discuss in detail the cancer-protective and cancer-promoting effects of NSAIDs, as well as other potentially lethal side effects of these drugs.

## 2. Chronic Inflammation and Cancer

In order to understand the role of NSAIDs in cancer, one must examine the link between chronic inflammation and carcinogenesis. The relationship between chronic inflammation and cancer was first hypothesized by Virchow more than a century ago in 1863. He observed that sites of chronic inflammation were the origin of cancer and that tissue injury and the associated inflammation caused by some irritants encouraged cell proliferation [2]. To date, such observation is backed by many epidemiologic and experimental studies. Many molecular targets and signaling pathways in apoptosis, cell proliferation, and angiogenesis are common to both inflammation and carcinogenesis. Dysregulation of these signaling pathways during chronic inflammation often leads to aberrant expression of proinflammatory genes, which play a role in malignant transformation [18].

Many cytokines act like a double-edged sword in tumor development, depending on the tumor microenvironment. Some of these cytokines, which exert antitumor effects, may induce cell transformation and malignancy during chronic inflammation [19]. Some examples of cytokines that are involved in inflammation and the tumor microenvironment include tumor-necrosis factor-*α* (TNF-*α*), interleukin-6 (IL-6), transforming growth factor *ß* (TGF-*β*), and interleukin-10 (IL-10) [18]. The link between cancer and chronic inflammation is further strengthened by the fact that many cancer cells express cytokines and chemokines, as well as their receptors, all of which are important in cell proliferation, angiogenesis, cell migration, and metastasis [20]. In addition to cytokines, other proinflammatory molecules such as inducible nitric oxide synthase (iNOS), reactive oxygen species (ROS), and nuclear factor kappa-light-chain-enhancer of activated B cells (NF-kB) are also upregulated in chronic inflammation [21].

It is now widely accepted that chronic inflammation is involved in carcinogenesis. The underlying aetiology for cancer development as a result of inflammation may be infectious or noninfectious in nature. For example, *Helicobacter pylori* infection is associated with gastritis, peptic ulcer disease, mucosa-associated lymphoid tissue (MALT) lymphoma, and gastric adenocarcinoma [22], whereas hepatitis B and C infections are associated with chronic hepatitis, liver cirrhosis, and hepatocellular carcinoma [23]. On the other hand, colorectal cancer is a serious complication of inflammatory bowel disease. It has been reported that patients with colitis have a two to eight times higher relative risk of colorectal cancer compared to the general population [24]. Another example in which chronic inflammation plays a role in tumorigenesis is the development of oesophageal adenocarcinoma, which has been shown to be associated with chronic irritation of the lower esophagus due to gastroesophageal reflux, giving rise to Barrett's oesophagus, dysplasia, and subsequently adenocarcinoma [25].

## 3. Nonsteroidal Anti-Inflammatory Drugs (NSAIDs) and Their Role in Cancer

Many drugs belong to the class of drugs known as the NSAIDs. Some examples of NSAIDs include ibuprofen, mefenamic acid, celecoxib, aspirin, and diclofenac. These drugs have one common property, i.e., their ability to block the enzyme cyclooxygenase (COX) or prostaglandin endoperoxide H synthase (PGHS), even though they are very diverse in their chemical structures. The role of NSAIDs in cancer is best viewed in the interrelationships between COX, prostaglandin synthesis, and inflammation.

### 3.1. COX, Prostaglandin Synthesis, and Inflammation

**Figure 1:**
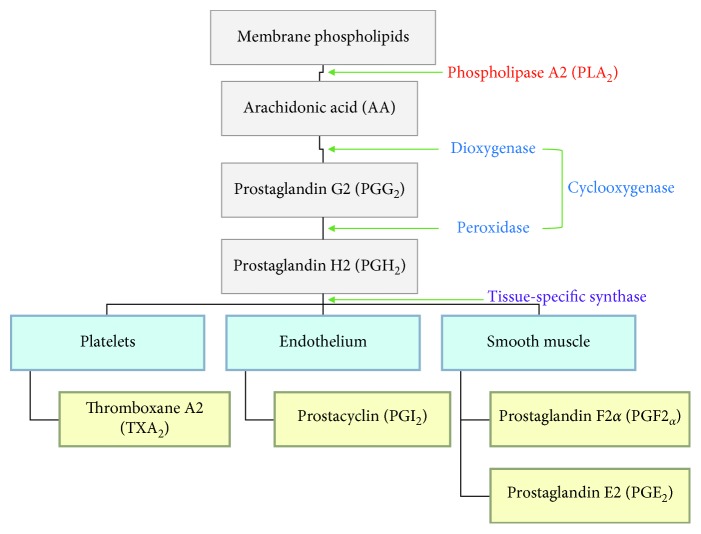
The arachidonic acid pathway and the role of cyclooxygenase in prostaglandin synthesis.

COX are enzymes that are involved in the synthesis of prostaglandins (PGs), which are derived from the arachidonic acid pathway (Figure 1). These COX-derived prostaglandins belong to a group of 20-carbon lipid compounds known as eicosanoids. They are widely found in the body with many physiological functions and are known mediators of inflammation. The synthesis of prostaglandin begins with the enzymatic action of phospholipase A2 (PLA_2_) on membrane phospholipids, which produces arachidonic acid (AA). AA is then metabolized to prostaglandins by COX in two steps. First, a dioxygenase activity acts on AA to produce prostaglandin G2 (PGG_2_) and subsequently, PGG_2_ is reduced to prostaglandin H2 (PGH_2_) by a peroxidase activity. On the other hand, tissue-specific synthases help synthesize PGE_2_, PGD_2_ PGF2α, PGI_2_, and thromboxane A2 (TXA_2_) from PGH_2_ [26].

During inflammation, PGE_2_ augments vasodilatation and increases microvascular permeability, which lead to the classical signs of redness and swelling. It also acts on the neurons of the sensory nervous system and gives rise to pain experienced during the inflammatory process [27]. On the other hand, PGI_2_ is a potent vasodilator and an inhibitor of platelet aggregation [28]. It is mainly produced by vascular, endothelial, and smooth muscle cells and is involved in the regulation of cardiovascular homeostasis, whereas PGD_2_ is the major eicosanoid synthesized in the central nervous system and peripheral tissues, which appears to play a role in both inflammation and homeostasis [29]. The research has shown that PGD_2_ is produced as the predominant prostanoid by activated mast cells and plays a role in the initiation of type I acute allergic responses mediated by immunoglobulin E (IgE) [30]. Another prostaglandin, PDF2_*α*_, is derived from COX-1 in the female reproductive system predominantly. Other than its involvement in ovulation, uterine contraction, and parturition initiation, PGF2_*α*_ has been found at sites of inflammation such as in the synovial fluid collected from the joints of patients with rheumatoid arthritis, psoriatic arthritis, osteoarthritis, and reactive arthritis [31].

It is worth mentioning that platelets are also active players in the inflammatory processes as a result of COX's activity on prostaglandin synthesis. Although platelets were once primarily recognized as a key player in haemostasis, its role in inflammation and cancer has been increasingly described in the published literature. TXA_2_ is a PGH_2_-derived substance produced by activated platelets, which exerts a potent vasoconstrictor effect and a stimulatory effect on platelet aggregation. However, other than its haemostatic role, TXA_2_ has been shown to be involved in inflammation and linked to allergic reactions, modulation of acquired immunity, angiogenesis, and cancer cell metastasis [32].

Platelets' influence on tumorigenesis may involve (i) enhancement of tumor cell survival by forming platelet aggregates surrounding tumor cells, (ii) increased tumor cell adhesion to the endothelium that leads to tumor cell arrest and extravasation, and (iii) production of lipid products such as TXA_2_ that enhances tumor vascularisation and dissemination of tumor cells into the bloodstream [33]. The past research has reported platelet-induced overexpression of COX-2 in human colon carcinoma cells [34], whereas increased COX-2-dependent PGE_2_ synthesis has been linked to tumorigenesis by mechanisms such as suppression of dendritic, natural killer, and T cells and type-1 immunity, as well as promotion of type-2 immunity that in turn promotes tumor immune evasion [35]. In addition, PGE_2_ was demonstrated to promote colorectal cancer stem cell expansion and metastasis [36].

### 3.2. Classification of NSAIDs Based on Their COX Interaction and Selectivity

The inhibitory actions of NSAIDs on COX have made these drugs popular targets in cancer prevention, in view of the close relationship between chronic inflammation and cancer. There are two isoforms of COX, i.e., COX-1 and COX-2. In general, both COX-1 and COX-2 are involved in prostaglandin synthesis. Prostaglandins produced by COX-1 play a role in platelet function and gastrointestinal cytoprotection, whereas those produced by COX-2 are involved in pain and inflammation [37]. The different functions of the COX isoforms help explain the differences in the therapeutic effects and side effects of various classes or subgroups of NSAIDs. There are several ways to categorize NSAIDs. One way of classification is based on the kinetics of their interaction with COX-1 or COX-2. Such interactions can be (i) freely reversible (e.g., piroxicam and ibuprofen), time-dependent, (ii) slowly reversible (e.g., diclofenac, indomethacin, and celecoxib), and (iii) irreversible (e.g., aspirin) (reviewed by Tacconelli et al.) [38].

Another way of classifying NSAIDs is based on their *in vitro* COX selectivity as the intrinsic ability of NSAIDs to inhibit COX-1 and COX-2 differs considerably. The determination of the IC_50_ (i.e., concentration at which 50% of COX activity is inhibited) of COX-1 and COX-2 *in vitro*, followed by the assessment of the ratio for COX-1 IC_50_ and COX-2 IC_50_, can be used to deduce the preferential COX selectivity of these drugs. A ratio of 1 indicates that the drug inhibits COX-1 and COX-2 to a similar extent. A ratio >1 indicates that the drug is preferentially selective toward COX-2, whereas a ratio <1 indicates that the drug is more selective for COX-1. For example, COX-1/COX-2 ratios for naproxen and ibuprofen have been reported to be 0.49 and 0.56, respectively, while NSAIDs that have a ratio >1 include acetaminophen (1.6), meloxicam (13.8), diclofenac (24.4), celecoxib (32), and etoricoxib (162) (reviewed by Tacconelli et al.) [38].

### 3.3. Differences in the Indications and the Therapeutic and Side Effects of NSAIDs

Although NSAIDs exert their anti-inflammatory, antipyretic, and analgesic effects through the inhibition of prostanoid biosynthesis, they differ variably in their indications and the therapeutic and side effects due to their differences in COX selectivity. While the therapeutic effects of NSAIDs can be largely attributed to COX-2 inhibition especially at the sites of inflammation, COX-1 inhibition is generally responsible for the many NSAID-associated side effects, particularly those related to the gastrointestinal (GI) tract [39]. Researchers have made many efforts to avoid the GI side effects through the development of selective COX-2 inhibitors known as the “coxibs” (e.g., celecoxib and etoricoxib), which aim to inhibit COX-2 while sparing COX-1. Therefore, COX-2 selectively can be viewed clinically as a variable that describes the probability of COX-1 sparing, which avoids the side effects of COX-1 inhibition at therapeutic doses of NSAIDs [40].

Another important difference in the therapeutic and side effects, as well as the indications of NSAIDs, is the extent to which platelet functions are affected by NSAIDs based on their COX selectivity. COX-1 and COX-2 are both inhibited by aspirin and the nonselective NSAIDs. COX-1 inhibition by aspirin and nonselective NSAIDs blocks TXA_2_ production, which interferes with normal platelet aggregation. This explains why low-dose aspirin, which irreversibly inhibits platelet aggregation, is indicated in the prophylaxis against ischaemic heart disease and stroke, whereas the COX-2 selective inhibitors are not used in such a manner because they have little or no effect on COX-1 [41]. As for the nonselective NSAIDs that reversibly inhibit COX-1, research has shown that variable, low levels of COX-1 inhibition may not be sufficient to provide cardioprotection when compared to aspirin's higher level of irreversible inhibition [42]. The cardiovascular risk of COX-2 inhibitors has been a debatable topic, and findings from various studies are contradictory. While some claim that there is no difference between traditional NSAIDs and the coxibs [43], others have reported an increased cardiovascular risk with the use of the latter [44].

It is also worth mentioning that some nonaspirin NSAIDs interfere with the antiplatelet effect of aspirin if these drugs were to be taken concomitantly. Earlier studies showed that taking ibuprofen 2 hours before aspirin affected the latter's antiplatelet effects [45], whereas sequential administration of naproxen with aspirin was shown to interfere with aspirin's irreversible inhibition of COX-1 with a smaller interaction observed when naproxen was given 2 hours after aspirin [46]. One study reported that celecoxib and other coxibs bind tightly to a subunit of COX-1 *in vitro* and interfered with COX-1 inhibition by aspirin. Administration of celecoxib in animals further showed an interference with aspirin's ability to inhibit platelet aggregation. These findings led to the inference that coxibs may blunt aspirin's cardioprotective effects [47].

Besides differences in the cardiovascular risks, differences in occurrence of GI adverse effects also exist for NSAIDs according to their COX selectivity. In general, NSAIDs with a greater selectivity for COX-2 have been associated with a lower occurrence of these adverse effects. In one study, there was a significantly lower rate of gastroduodenal ulcer for celexocibe (4%) when compared to naproxen (19%, *p* < 0.001)after a 0- to 4-week interval. For the 4- to 8-week interval, the rates were 2% versus 14% (*p* < 0.001) and the 8- to 12-week interval, 2% vs 10% (*p* < 0.001). Overall, a significantly lower gastric ulcer (*p* < 0.001) and duodenal ulcer (*p* < 0.030) rate was observed for celecoxib when compared to naproxen. However, both drugs were comparable in their efficacy in patients with osteoarthritis and rheumatoid arthritis [48]. It is worth noting that concomitant use of aspirin and a COX-2 selective inhibitor such as rofecoxib (16.1%) had a significantly higher ulcer incidence when compared to the use of aspirin alone (7.3%, *p* < 0.001), but such an increase in incidence was no less than that associated with the use of a nonselective NSAID such as ibuprofen (17.1%) as reported in another study [49].

### 3.4. Targeting Inflammation in Cancer

Due to the relation between chronic inflammation and cancer, it is reasonable for researchers to target inflammation in cancer treatment and prevention. Some targets that have been explored in combating inflammation relating to cancer include COX, NF-kB, cytokines/chemokines and their receptors, and fibroblast growth factor (FGF) and its receptor, as well as vascular endothelial growth factor [50]. It is worth mentioning that COX was found to be overexpressed in various cancers such as pancreatic [51], prostate [52], cervical [53], breast, lung, and colon [54] cancer in the past few decades. The overexpression of COX, in turn, was found to stimulate angiogenesis [55], which is a key step in invasion and metastasis. The overexpression of COX was also reported to be precancerous, by increasing the resistance of cancer cells to apoptosis in one earlier study [56].

As NSAIDs are well-known COX inhibitors, they are inevitably a popular anticancer anti-inflammatory candidate in cancer therapy and prevention. Inhibiting COX may be seen as a good strategy because some of the products of COX activity (e.g., prostaglandin E_2_) are involved in tumorigenesis. Prostaglandin E_2_ (PGE_2_) was shown to be increased in cancer cells [57] and is capable of stimulating cancer cell proliferation and invasion [58]. The COX-2/PGE_2_ signaling pathway has been reported to play a crucial role in colorectal tumorigenesis. Research suggests that an increase in the expression of COX-2 and PGE_2_ supports colorectal cancer cell survival especially in a glucose-deprived tumor microenvironment [57].

## 4. Role of NSAIDs in Cancer Prevention

Data on the cancer-protective effects of NSAIDs are abundant and overwhelming in the published literature. There is much epidemiologic and experimental evidence that points to the antitumor effects of NSAIDs in many types of cancer. This section will discuss the role of NSAIDs in chemoprevention using evidence from *in vitro*, *in vivo*, and epidemiologic studies. In some of these studies, a difference was observed between aspirin and nonaspirin NSAIDs, while others showed no clear distinction between the two.

### 4.1. Evidence from In Vitro and In Vivo Studies

Many experimental studies have explored the underlying mechanisms of anticancer effects of NSAIDs either using cell lines or animal models. Several nonaspirin NSAIDs such as celecoxib [59] and loxoprofen [60] have been shown to exert their cancer-protective effects. For example, in an *in vivo* study using a rat mammary model, celecoxib demonstrated a 90% tumor regression and a 25% reduction in the number of palpable tumors [59]. Another study revealed that ibuprofen-inhibited cell proliferation in mouse and human colorectal cells. A 40%–82% tumor growth inhibition and a reduction in liver metastases in mice with colorectal cancer were also observed in the same study [60]. On the other hand, loxoprofen was demonstrated to inhibit the growth of implanted Lewis lung carcinoma in mice. Mice treated with the drug showed a significant lower intratumoral vessel density and mRNA expressions of vascular endothelial growth factor (VEGF) in the tumor, whereas in the same study, the plasma levels of VEGF in non-small cell lung cancer patients treated with loxoprofen (120 mg/day) for one week were also shown to be significantly reduced. Findings of the study point to possible suppression of angiogenesis through the inhibition of VEGF [61].

Similarly, studies have also reported the anticancer effects of aspirin. Xiang et al. demonstrated the antiapoptotic and antiproliferative effects exerted by aspirin on HeLa cells. A time- and dose-dependent reduction of ErbB2 expression was observed in these cervical cancer cells, whereas the underlying mechanism of aspirin's antiapoptotic effects was due to its inhibition on the activation of extracellular signal-regulated kinase (ERK) and AKT (also known as protein kinase B), as well as the inhibition of Bcl-2 expression [62]. In another study, aspirin was shown to have synergic anticancer effects on HepG2 human hepatocellular carcinoma when combined with doxorubicin both *in vivo* and *in vitro*. A strong synergism was observed in cell-cycle arrest, growth inhibition, and apoptosis *in vitro* when the two drugs was used in combination, whereas a synergic antitumor activity was observed in nude mice with a HepG2 cell xenograft [63].

Interestingly, many of the anticancer effects of NSAIDs are often independent of COX inhibition. These COX-independent mechanisms are explained by the fact that NSAIDs possess antiproliferative and apoptotic effects on cell lines regardless of their level of COX expression [64, 65]. The fact that the growth-suppressing effects of NSAIDs in cancer are not reversible with prostaglandin supplementation further suggests that NSAIDs work through COX-independent mechanisms in cancer suppression [66]. In one study, indomethacin was demonstrated to induce apoptosis in esophageal adenocarcinoma cells, in which the underlying mechanisms involved COX-2-independent Bax upregulation and mitochondrial cytochrome C translocation [64]. Another study revealed COX-2-independent NSAID-induced apoptosis in malignant melanomas [65]. Other NSAID COX-independent strategies in cancer therapy include modulation of cGMP phosphodiesterase signaling, inhibition of NF-κB signaling, inhibition of AMP-activated protein kinase, induction of PPARγ promoter activity, suppression of Akt signaling, and inhibition of metastasis and angiogenesis (reviewed by Gurpinar et al.) [67].

### 4.2. Evidence from Epidemiologic Studies

There are numerous epidemiologic studies that examined the cancer-protective effects of both aspirin and nonaspirin NSAIDs. Among these studies, many have been done on cancers of the gastrointestinal tract. In an earlier study that investigated the relation between NSAID use (which included both aspirin and nonaspirin NSAIDs) and digestive cancers other than colorectal cancer, it was found that the risk for gastric cancer was reduced in regular NSAID users (OR 0.3; 95% CI: 0.1–0.6) [68]. In another study that involved 10,280 cases and 102,800 controls, the association between colorectal cancer risk and the use of aspirin and NSAIDs was investigated. A 27% decrease in colorectal cancer risk was observed in low-dose aspirin use (OR = 0.73; 95% CI: 0.54 to 0.99). For the nonaspirin NSAID users, a substantial reduction in risk was observed especially for those who used agents with high COX-2 selectivity on a long-term, high-intensity basis (OR = 0.57; 95% CI: 0.44 to 0.74) [9].

In the Sister Study, which investigated women with a sister who had breast cancer, there were 2118 incident breast cancers from 50,884 women enrolled in the study. It was observed that the use of nonaspirin, noncoxib NSAIDs was not associated with a reduced breast cancer risk among postmenopausal women. However, for the premenopausal women, there was a reduction in breast cancer risk for any nonaspirin NSAID (HR_4vs1_ = 0.66, 95% CI: 0.50–0.87) and for aspirin specifically (HR_4vs1_ = 0.57, 95% CI: 0.33–0.98). The study concluded that those with increased breast cancer risk such as having a sister with the disease might benefit from using NSAIDs as a mean of chemoprevention [4].

A case control study of 1736 breast cancer cases and 1895 health controls in Spain reported similar findings with a 24% reduction in breast cancer risk (OR = 0.76; 95% CI: 0.64–0.89) in those who used NSAIDs. However, such reduction in risk was not observed with those who used aspirin. The findings were similar for postmenopausal and premenopausal women. It is important to note that the protective effects of NSAIDs in breast cancer only applied to certain subtypes in this study. The protection was seen in hormone + or HER2+ cancers but not applicable to triple-negative breast cancers [5]. Findings of this study are in tandem with those of another earlier study, which reported that NSAIDs' protective effects on breast cancer were dependent on the molecular subtypes, in which there was an increased risk observed in certain subtypes and a decreased risk in others [15].

Other than the cancers arising from the gastrointestinal tract and breast cancer, NSAIDs were associated with a reduced risk of cancers originating from the reproductive system in both sexes. In men, NSAIDs were reported to be associated with a reduction in prostate cancer risk in a study that investigated 819 prostate cancer patients and 879 controls. All NSAIDs were inversely associated with a reduction in prostate cancer risk (OR = 0.77, 95% CI: 0.61–0.98), especially for drugs that preferentially inhibit COX-2 (OR = 0.48, 95% CI: 0.28–0.79). A reduced risk was also observed in men with aggressive prostate cancer using nonaspirin NSAIDs (OR = 0.49, 95% CI: 0.27–0.89) and nonaspirin users with a history of prostatitis (OR = 0.21, 95% CI: 0.07–0.59). In the same study, aspirin use was slightly and negatively associated with prostate cancer (OR = 0.86, 95% CI: 0.65–1.14); however, such association was not statistically significant (*p* > 0.05) [7]. In another study, a decreased cancer risk was observed in women with ovarian cancer in using pooled data from 12 population-based studies with 7776 cases and 11843 controls. Aspirin, but not NSAIDs, was reported to be associated with a reduced risk of ovarian cancer (OR = 0.91; 95% CI: 0.84 to 0.99) [10].

Rothwell et al. followed up four randomized trials (i.e., Thrombosis Prevention Trial, British Doctors Aspirin Trial, Swedish Aspirin Low-Dose Trial, and UK-TIA Aspirin Trial) on the long-term effect of aspirin on the incidence and mortality of colorectal cancer for 20 years. The scheduled treatment duration was 6 years, and the median of the follow-up duration was 18.3 years. Of 14033 patients, 391 (2.8%) had colorectal cancer. Findings showed that there was a significant reduction in the 20-year risk of colon cancer (incidence hazard ratio (HR) 0.76, 0.60–0.96, *p*=0.02; mortality HR 0.65, 0.48–0.88, *p*=0.005) but not rectal cancer (0.90, 0.63–1.30, *p*=0.58; 0.80, 0.50–1.28, *p*=0.35). However, it was reported that there was no additional benefit for aspirin doses >75 mg daily or a duration >5 years of scheduled treatment with 75–300 mg of aspirin daily [69]. This is supported by evidence from two large studies, i.e., Nurses' Health Study (NHS, 1980–2010) and Health Professionals Follow-Up Study (HPFS, 1986–2012), which reported a reduction in overall cancer risk (RR 0.97; 95% CI: 0.94, 0.99), primarily due to a lower incidence of GI cancers, especially colorectal cancers [70].

## 5. Role of NSAIDs in Cancer Promotion

Compared to studies on the cancer-protective effects of NSAIDs, there are relatively fewer studies on the risk-enhancing effects of NSAIDs in cancer. Studies that reported NSAIDs' role in increasing cancer risk are mostly epidemiologic, and the mechanisms underlying the increased risk are less well delineated. There have been several reports on the association between NSAID use and increased risk of renal cancer. In an earlier study that followed up 77,525 women for 16 years and 49,403 men for 20 years, 333 renal cell carcinoma cases were documented. A dose-response relation was observed between duration of nonaspirin NSAID use and renal cell carcinoma risk. For users <4 years, 4–10 years, and >10 years, the relative risks (RR) were 0.81 (95% CI: 0.59–1.11), 1.36 (95% CI: 0.98–1.89), and 2.92 (95% CI: 1.71–5.01), respectively (*P*_trend_ < 0.001) [71].

In another study, a meta-analysis of epidemiologic studies concerning analgesic use and kidney cancer risk revealed an increased risk of kidney cancer with the use of acetaminophen (pooled RR, 1.28; 95% CI: 1.15 to 1.44) and nonaspirin NSAIDs (pooled RR, 1.25; 95% CI: 1.06 to 1.46). However, there was no overall increased risk in those who used aspirin (pooled RR, 1.10; 95% CI: 0.95 to 1.28) [16]. One possible explanation for such association is that NSAIDs can lead to acute and chronic renal injury, which may theoretically lead to carcinogenesis. However, further exploration is required to unfold the underlying mechanisms in tumor development.

Although earlier studies reported that the use of NSAIDs reduces the risk of endometrial cancer [72, 73], the role of NSAIDs in endometrial cancer remains unclear as there are some conflicting findings in this area of research. In a more recent study that investigated the relationship between NSAIDs and endometrial cancer mortality and recurrence, an association (HR = 1.66, 95% CI: 1.21 to 2.30) was observed between NSAID use and increased endometrial carcinoma-specific mortality in type I cancer. A significant association was observed among both current and former users, with the strongest association seen in former users for ≥10 years (HR = 2.23, 95% CI: 0.19 to 4.18, two-sided *P*_trend_=0.01). However, such an association was not observed in women with type II endometrial carcinoma [74].

For breast cancer, many studies have demonstrated an inverse relationship between NSAID use and cancer risk [3–5]. However, it is worth mentioning that such a relationship depends on the molecular subtype of breast cancer. In Western New York, a population-based case-control study (*n* = 1170) showed that an increased risk of ER+/PR+ (OR = 1.33, 95% CI: 1.09–1.62), HER2− (OR = 1.27, 95% CI: 1.05–1.53), and p53− breast cancers (OR = 1.28, 95% CI: 1.04–1.57) was associated with ibuprofen use [15]. These findings are in tandem with an earlier study (Nurse's Health Study II) in which 2-3 times per week nonaspirin NSAID use was associated with increased breast cancer risk (RR = 1.37, 95% CI: 1.09–1.67), but the hormone receptor status did not play a role in this study [75]. The underlying mechanisms of this association and increased risk of breast cancer are not clear and warrant further exploration.

In one study, an elevated prostate cancer risk among current NSAID users was observed in the screening (HR = 1.45, 95% CI: 1.33–1.59), as well as the control (HR = 1.71, 95% CI: 1.58–1.86) groups, and the risk was similar for coxib and acetaminophen current users. It is worth mentioning that a stronger risk was observed for metastatic prostate cancer for subjects in both the screening (HR = 2.41, 95% CI: 1.59–3.67) and control (HR = 3.44, 95% CI: 2.60–4.55) groups. However, the study concluded that the increased risk in prostate cancer was not directly caused by the medication as it was observed only for ongoing prescription use and was the strongest for subjects with metastatic disease, and that the risk was not affected by the amount or duration of NSAID usage [76].

It is important to note that aspirin and NSAIDs are good painkillers and both drugs lower prostate specific antigen (PSA) levels [77]. This may mean masking of symptoms and a delay in diagnosis. Hence, many of the studies that report a reduced risk in the published literature need to be carefully evaluated. Epidemiologic studies may be suggestive but are not conclusive, which warrant a more detailed investigation of the mechanisms behind the effects of NSAIDs in cancer.

Just like the nonaspirin NSAIDs, aspirin has also been reported to increase the risk of cancer. In a recent large, single-centre cohort study which based on data from the Northwestern Medicine Enterprise Data Warehouse, all patients without malignant melanoma from 18 to 89 years were followed up for 5 years after once-daily aspirin for ≥1 year. The findings suggested that there was an increased overall risk for malignant melanoma with once-daily aspirin exposure in a dose-dependent manner, particularly in males. However, the underlying mechanisms for these findings are unclear. It is also worth mentioning that the study was limited by the inability to verify certain information, such as the adherence to aspirin consumption, history of sun exposure, and the skin phototype of the patients [78].

Nevertheless, as there exist conflicting views regarding the effects of chronic aspirin use in melanoma, whether aspirin increases or decreases the risk of melanoma remains controversial. In a recent study by Kumar et al. [79], the inhibitory effects of aspirin were explored using melanoma and melanocyte cell lines, as well as an animal model. The study showed that aspirin and celecoxib significantly decreased cell motility, colony formation, and melanin production *in vitro*. It was further demonstrated that a reduction in both melanoma tumor growth and proliferation was observed in NOD/SCID mice with chronic daily aspirin use. Melanoma tumor-xenografted mice treated with aspirin exhibited decreased PGE_2_ levels in plasma and tumors and increased 5′-adenosine monophosphate-activated protein kinase (AMPK) in tumors. Given the contradictory findings from different studies, further exploration is needed before any conclusive remarks can be made on the role of aspirin in melanoma.

## 6. Noncancerous, Life-Threatening Side Effects of NSAIDs

Although this review focuses on the role of NSAIDs in cancer, not to be forgotten are the noncancerous but serious side effects of NSAIDs. It is generally accepted that NSAIDs are associated with an increased risk of acute myocardial infarction. Recently, it was reported that myocardial infarction risk was associated with NSAID use. The drugs investigated were celecoxib (OR = 1.24; 95% CI: 0.91–1.82), ibuprofen (OR = 1.48; 95% credibility interval (CI):1.00–2.26), diclofenac (OR = 1.50; 95% CI: 1.06–2.04), naproxen (OR = 1.53; 95% CI: 1.07–2.33), and rofecoxib (OR = 1.58; 95% CI: 1.07–2.17) [80].

In a meta-analysis examining the cardiovascular safety of NSAIDs, 31 trials consisting of 116, 429 patients were included. The cardiovascular risk of naproxen, ibuprofen, diclofenac, celecoxib, etoricoxib, rofecoxib, and lumiracoxib was examined. It was found that the highest myocardial infarction risk was associated with rofecoxib (rate ratio = 2.12, 95% credibility interval (CI): 1.26–3.56), followed by lumiracoxib (rate ratio = 2.00; 95% CI; 0.71–6.21). As for stroke, the highest risk was associated with ibuprofen (rate ratio = 3.36, 95% CI: 1.00–11.6), while diclofenac was associated with the second highest risk (rate ratio = 2.86, 95% CI: 1.09–8.36). On the other hand, two drugs were associated with the highest risk of cardiovascular death, i.e., etoricoxib (rate ratio = 4.07, 1.23–15.7) and diclofenac (rate ratio = 3.98, 95% CI: 1.48–12.7). The study concluded that there was little evidence that suggested the drugs investigated were safe with respect to the cardiovascular system and that naproxen appeared to be the least harmful among them [81].

Many NSAID users experience gastrointestinal side effects ranging from nausea, mild discomfort, and dyspeptic symptoms to severe complications such as bleeding, peptic ulcer perforation, and intestinal obstruction (reviewed by Sostres et al.) [82]. Common and important risk factors for developing GI adverse effects in NSAID users include a past medical history of peptic ulcer disease, age, and concomitant use of aspirin [83]. One meta-analysis reported the association of increased risk of gastrointestinal complications with 16 different types of NSAIDs. The relative risks were between 2 and 4, with ketorolac and azapropazone showing the highest risk (RR = 11.5 and 18.5, respectively), whereas aceclofenac and celecoxib had the lowest risk (RR = 1.4 and 1.5, respectively). It was also observed that the risk increased with increasing NSAID dosage [48]. Patients who develop GI complications are at risk of dying. Although the mortality rate for upper GI bleed and peptic ulcer perforation have decreased over time in the past few decades in the general population, the mortality rate for those using NSAIDs or aspirin remains high, with about one in five who develop upper GI bleed or peptic ulcer perforation dying from such complications [84].

In addition to serious cardiovascular and gastrointestinal side effects, NSAIDs are well recognized for their renal side effects, which may lead to renal failure in severe cases. Previous studies have reported an increased risk in acute renal failure. One study reported a threefold increased risk when comparing NSAID users and non-NSAID users (95% CI: 1.8–5.8) [85], while another reported a relative risk of 2.30, 2.31, and 2.42 for traditional NSAIDs, rofecoxib, and naproxen, respectively. The latter also revealed that the increased risk of acute renal failure was dose dependent [86].

## 7. Conclusions

Several conclusions can be drawn from this review. Firstly, there are numerous studies on the use of NSAIDs in cancer, which include both aspirin and nonaspirin NSAIDs. These studies show inconsistent and contradicting findings in terms of the role of these drugs in cancer, with some of them reporting an increased risk in certain types of cancer and others showing a reduction in cancer risk. Secondly, many of these studies are epidemiologic in nature, although there are also some experimental studies that examined the underlying mechanisms of the cancer-protective effects of NSAIDs. Therefore, the mechanisms underlying these effects are not well understood, especially in studies that claimed an increase in cancer risk. Epidemiologic studies are often suggestive but not conclusive, which implies that more experimental studies are needed in this area of research. Thirdly, whether NSAIDs increase or decrease the risk of cancer depends on the type of cancer. Even within the same cancer type, the effects of NSAIDs may vary between different molecular subtypes, as seen in the example of breast cancer. Lastly, NSAIDs are associated with other noncancerous, serious complications such as myocardial infarction, gastrointestinal bleeding, and renal failure. As a result, the use of NSAIDs in cancer treatment and prevention is to be assessed with much caution and there must be a balance between the risks and the benefits in view of the inconsistent findings, not-well-understood underlying mechanisms, and potentially life-threatening side effects.
